# Midwives’ knowledge and diagnostic practices for mastitis and breast cancer in breastfeeding women in Japan: A cross-sectional study

**DOI:** 10.18332/ejm/209494

**Published:** 2025-08-29

**Authors:** Yuki Kanazawa

**Affiliations:** 1 Faculty of Nursing and Medical Care, Keio University, Fujisawa, Japan

**Keywords:** breast neoplasms, lactation, mastitis, midwifery, breastfeeding, pregnancy

## Abstract

**INTRODUCTION:**

Japanese midwives support lactating women to continue breastfeeding. However, midwives often learn breast care methods through practical experience. This study investigated how midwives acquire knowledge about mastitis and breast cancer.

**METHODS:**

This cross-sectional study was conducted in Japan over two months. The study participants were midwives with breast care experience. The questionnaire was sent to 800 midwifery facilities for recruitment. The questions covered learning and diagnostic methods for general breast care, bacterial mastitis, severe mastitis, and breast cancer during lactation. The analysis method involved descriptive statistics. An Ethical Review Committee approved this study.

**RESULTS:**

The survey return rate was 27.50% (n=200). The valid response rate was 87.27% (n=192). Although the learning method that helped midwives most regarding mastitis, in general, was breast care experience (38.0%), knowledge about bacterial mastitis and severe mastitis came from advice from doctors or senior midwives (33.3%, 42.4%). However, knowledge about breast cancer during lactation was mostly learned during formal education (29.5%); many had never learned about it (10.5%). The most common method used by midwives to make breast care decisions was subjective judgment.

**CONCLUSIONS:**

Most midwives learned a great deal by observing and palpating actual breasts in clinical settings. Some midwives had learned very little about breast cancer. Most midwives did not use medical equipment for breast evaluations. This suggests that Japanese midwives have high breast care skills. However, there is room for improving midwives' skills in using medical equipment.

## INTRODUCTION

In Japan, approximately 80–90% of mothers wish to breastfeed^1^. Globally, mothers’ desire to breastfeed is high; however, 52.59% of mothers who received breastfeeding support could not continue breastfeeding by six months^2^. First, breastfeeding stabilizes maternal mental health^3,^ ^4^. However, even if women wish to breastfeed, they may be unable to continue breastfeeding, which may cause postpartum depression. Recently, postpartum maternal suicides have increased owing to an increase in postpartum depression^5-7^.

Second, oxytocin secreted during breastfeeding promotes social interactions and attachment formation between mothers and children^8^. However, breastfeeding is difficult for some women. When low breast milk volumes are secreted, children become malnourished, increasing the required feeds per day and leading to maternal sleep deprivation and fatigue^9^. If too much milk is produced, breasts can become swollen or painful and develop mastitis. If the child cannot suckle properly, the nipple is damaged, causing maternal pain. The breast structure influences breastfeeding^10^. Therefore, when women have difficulty breastfeeding, they often cannot continue breastfeeding.

Third, unless mothers request it, few points of contact exist. During maternal hospitalization, midwives guide mothers on childcare issues, such as breastfeeding, bathing, and changing diapers. After hospital discharge, midwives contact patients by phone; two weeks and 1 month after giving birth, mothers and children can visit the hospital for medical examinations and advice from midwives regarding childcare. Mothers can also consult midwives and community-based public health nurses about childcare. However, if women do not feel they need midwife-led breastfeeding care or are unaware of its existence, they will not connect with midwives. Therefore, Japanese women frequently cannot continue breastfeeding.

Midwifery qualifications in Japan are not renewed after graduation. Midwives are required to complete continuing education after obtaining their licenses. Furthermore, most schools that offer midwifery training do not teach breast massage techniques, specific diagnostic methods, or breast cancer diagnostics. Japan has published mastitis care guidelines^11^, which include a flowchart for identifying and differentiating mastitis.

Finally, midwives support lactating women to continue breastfeeding. However, they most likely learn how to provide this support through practical experience. Midwives can be divided into those who try to attend training sessions and those who do not make an effort towards further education or think it is unnecessary. Therefore, this study aimed to survey how midwives who have obtained midwifery qualifications acquire knowledge about mastitis and breast cancer. This study also provides suggestions for educational methods to solidify and improve breast knowledge in midwives after obtaining midwifery qualifications.

## METHODS

### Study design and setting

This cross-sectional study was based on a survey using an anonymous self-administered questionnaire. The study setting included 47 prefectures in Japan. The survey was sent out on 21 December 2023, with 28 January 2024 as the return deadline.

### Participants

The participants included midwives with experience in providing breast care. The study excluded nurses and midwives who provided breast care as nurses before obtaining their midwife certifications and did not provide breast care after obtaining their midwife certifications. In Japan, only women can obtain the midwife certification. Therefore, this study did not include men. The required participant number for the survey was calculated as:

n=Z^2^p(1-p)/d^2^

where p is the expected prevalence (0.5), d is the margin of error (0.05), and Z statistic equal to 1.96 for 95% level of confidence, giving a sample size n=384. Regarding the sampling method, we randomly selected 800 midwifery facilities whose addresses were listed on each prefectural midwifery association’s website and mailed each facility a request for research cooperation.

### Ethical considerations

The questionnaire had a consent box; only those who checked that box were deemed to have agreed to study participation. As this study used an anonymous self-administered questionnaire survey, consent could not be withdrawn once the participant had dropped the questionnaire off at the post office. Approval was obtained from Ethics Review Board of Keio University (notification number 340JIN; approval date: November 24, 2023).

### Data sources and measurements

The questionnaire was developed in a previous study^12^; three researchers with midwifery qualifications carefully examined and revised it. The experts revised the questions and pointed out necessary and unnecessary questions. This study did not involve a pilot study. The participants were asked to place the completed questionnaire into a collection envelope and drop it into a post office mailbox.

### Variables in the survey and their integration in the analysis

#### Mastitis care question variables and their integration in the analysis

The variables of mastitis care question in the survey were: 1) number of mild and severe mastitis cases treated; 2) the most important learning method regarding mild and severe mastitis diagnosis and treatment (the items were: reading books, participating in training sessions, browsing websites, during care experiences, advice from doctors or senior midwives, while enrolled as midwifery students, schools specializing in breastfeeding care, never learned about it, and other); 3) number of infectious mastitis cases treated; 4) the most important way to learn about infectious mastitis diagnosis and treatment (the items were as for variable 2); 5) number of severe mastitis cases (those with purulent discharge) treated; 6) the most profound way to learn about infectious mastitis diagnosis and treatment (the items were as for variable 2); 7) the most utilized methods for diagnosing mastitis [the items were: medical history (patient’s complaints of pain, fatigue, fever, etc.), visual examination (breast conditions, such as redness), palpation (conditions that can be palpated, such as heat, lumps, etc.), use of the ‘Flow of differential diagnosis’ route according to the Mastitis Care Guidelines^11^ (Supplementary file Material 1), breast ultrasound examination, requesting a doctor to perform a breast ultrasound examination, requesting a doctor to perform a milk culture; and other]; 8) the most used method to differentiate between different mastitis types after a diagnosis of mastitis [the items were: differentiate by breast massage, use the mastitis care guideline ‘Mastitis Care Flowchart 2020’ (Supplementary file Material 1)^11^, differentiate by self-examination, differentiate by ultrasound examination by a doctor, differentiate by milk culture by a doctor, not particularly aware of differentiating between different mastitis types, and other]; and 9) please freely write the method used to identify the possibility of infectious mastitis before consulting a doctor. The actual survey content is provided in Supplementary file Material 2.

Therefore, some items regarding the mastitis diagnostic methods were integrated: 1) medical history, 2) visual examination and 3) palpation were classified as ‘one of the following: medical history, visual examination, or palpation’; 4) medical history and visual examination, 5) medical history and palpation and 6) visual examination and palpation were classified as ‘two of the following: medical history, visual examination, or palpation’ ; 7) medical history, visual examination, and palpation were maintained without integration; 8) use of the ‘Flow of differential diagnosis’ route according to the Mastitis Care Guidelines was maintained without integration; 9) medical history, visual examination, palpation, and ultrasound examination and 10) medical history and breast ultrasound examination were classified as ‘medical history, visual examination, palpation, and breast ultrasound’; 11) breast ultrasound examination was maintained without integration; 12) requesting a doctor to perform a breast ultrasound examination and 13) requesting a doctor to perform a milk culture were classified as ‘ask a doctor for a breast ultrasound or milk culture’.

#### Breast cancer questionnaire variables during lactation and their integration in the analysis

The questionnaire variables regarding how to identify and respond to breast cancer during lactation were: 1) number of times they had dealt with breast cancer during lactation; 2) the most important way to learn about how to identify and deal with breast cancer during lactation [the items were as for the mastitis care question variable 2)] ; and 3) the most suitable method for diagnosing breast cancer during lactation [the items were: medical history (patient’s complaints of lumps, discomfort, etc.), visual examination (condition of the breasts, such as differences between left and right), palpation (condition that can be palpated, such as lumps), breast ultrasound examination, requesting a doctor to perform a breast ultrasound examination, requesting a doctor to perform a milk culture, size of the lump after milk removal, when the lump recurs, using the ‘Flow of differential diagnosis’ route in the mastitis care guidelines^11^, requesting an obstetrician or technician to perform a breast ultrasound examination, when a lump is found recommending a visit to a breast clinic in any case and explaining the risk of breast cancer to the patient, and other].

Breast cancer variables during lactation were combined as follows: 1) medical history, 2) visual examination, and 3) palpation were classified as ‘one of the following: medical history, visual examination, or palpation’; 4) medical history and visual examination, 5) medical history and palpation and 6) visual examination and palpation were classified as ‘two of medical history, visual examination, and palpation’; 7) medical history, visual examination, and palpation were maintained without integration; 8) medical history, visual examination, palpation, and breast ultrasound examination and 9) breast ultrasound examination were classified as ‘breast ultrasound by myself’; 10) requesting a doctor to perform a breast ultrasound examination, 11) requesting a doctor to perform a milk culture, and 12) requesting an obstetrician or technician to perform a breast ultrasound examination were classified as ‘ask other medical staff for a breast ultrasound or milk culture’; 13) size of the lump after milk removal, 14) when the lump recurs, 15) when a lump is found recommending a visit to a breast clinic in any case and 16) when a lump is found explaining the risk of breast cancer to the patient were classified as ‘size of the lump after milk removal or when the lump recurs’; and 17) using the ‘Flow of differential diagnosis’ route according to the mastitis care guidelines was maintained without integration.

#### Variables of questions regarding participants’ demographics

The variables were: 1) age; 2) years of midwifery experience; 3) years of experience providing breast care (starting from when they were entrusted with breastfeeding care alone, such as in a breastfeeding clinic); 4) places where they learned about breastfeeding care (the items were: midwifery school, hospitals they worked at, Oketani Breast Milk Management Training Center^13^, Mama’s Breast Care technical training, and other); 5) current affiliation [the items were: midwifery clinic specializing in breastfeeding, midwifery clinic that handles childbirth, concurrent obstetric clinic and midwifery clinic, concurrent general hospital and midwifery clinic, general hospital, obstetric clinic, and other).

### Statistical analysis

As this study was a factual survey, descriptive statistics were conducted. First, the mean and standard deviation were calculated for the study participant characteristics. The median, minimum, and maximum values were calculated for the number of cases of treatment of mastitis, infectious mastitis, and severe mastitis. The ratios of each item were calculated and compared for the diagnosis and treatment of mastitis, infectious mastitis, and breast cancer during lactation. Second, regarding the mastitis diagnostic methods, the ratios of each item related to the method of identifying mastitis were calculated and compared. Third, the method of distinguishing mastitis type after being diagnosed with mastitis was calculated by the median, minimum, and maximum values.

Finally, the questionnaire variables regarding how to identify and respond to breast cancer during lactation were summed for each item; proportions were calculated and compared.

IBM SPSS Statistics 29.0. (IBM SPSS Inc., Armonk, NY, USA) was used for statistical analyses.

## RESULTS

**Figure 1:**
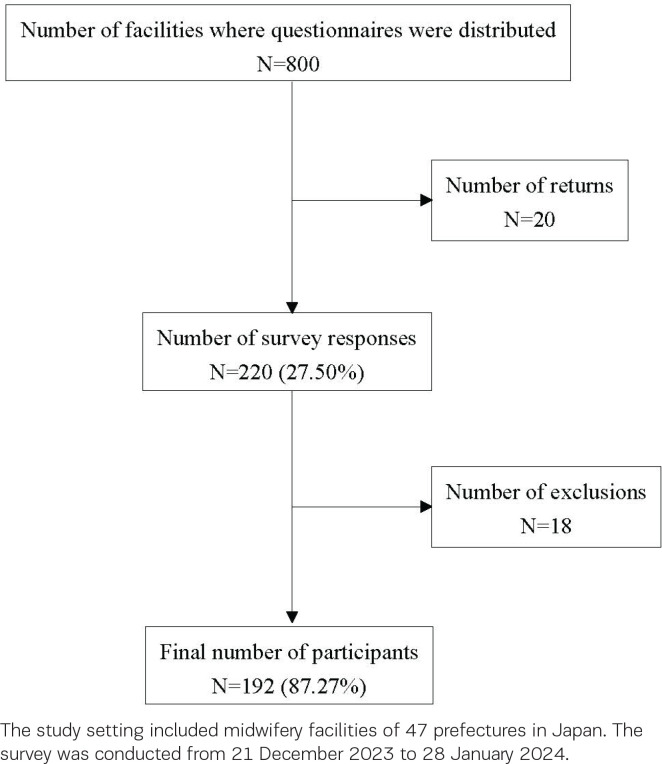
Participants flow diagram of Japanese midwives with experience in providing breast care

Questionnaires were sent to 800 facilities. There were no deliveries to 20 facilities because their addresses had changed. The questionnaire return rate was 27.50% (n=200). The valid response rate was 87.27% (n=192). Questionnaires with invalid responses were marked as missing (n=8). Figure 1 shows the participant flow diagram.

### Characteristics

The mean participant age was 54.07 ± 11.45 years. The number of years of providing breast care was 18.15 ± 10.33 years. Many study participants were affiliated with midwifery clinics specializing in breastfeeding. Table 1 presents the participant demographic characteristis.

**Table 1:** Participant demographic characteristics of Japanese midwives with experience in providing breast care (N=192)

*Characteristics*	*Mean ± SD (n)*
**Age** (years)	54.07 ± 11.45 (192)
**Years of experience as a midwife**	28.20 ± 11.51 (192)
**Years of providing breast care**	18.15 ± 10.33 (192)
**Affiliation**	
Midwifery clinic specializing in breastfeeding	39.06 (75)
Midwifery clinic that handles childbirth	27.08 (52)
Double-affiliation, obstetric hospital with a doctor and midwifery clinic	13.02 (25)
Obstetric hospital with a doctor	2.60 (5)
Other	18.23 (35)

The study setting included midwifery facilities of 47 prefectures in Japan. The survey was conducted from 21 December 2023 to 28 January 2024.

### Learning methods used to obtain knowledge about lactating breasts

The participants in this study indicated that the most useful learning methods for learning about mastitis were care experiences (38.0%) and advice from a doctor or senior midwife (22.9%). Knowledge about bacterial mastitis was primarily gained via advice from doctors and senior midwives (33.3%) and care experiences (24.5%). Knowledge about severe mastitis was principally obtained from advice from doctors and senior midwives (42.4%) and care experiences (20.9%). Knowledge about breast cancer during lactation was primarily obtained while enrolled as midwifery students (29.5%) and attending training sessions (25.3%). Some responded: ‘I have never learned anything’. Most respondents had never learned about breast cancer during lactation, followed by those who had not learned about severe mastitis (10.5% and 2.1%, respectively). Table 2 lists the proportion of participants who learned by each method.

**Table 2:** The most commonly used study method for Japanese midwives to learn about breastfeeding (N=192)

*Study method*	*Mastitis in general %*	*Bacterial mastitis %*	*Severe mastitis %*	*Breast cancer %*
Reading books	3.7	6.8	7.3	14.7
Participating in training sessions	19.8	19.8	15.2	25.3
Visiting websites	0.5	0.0	0.0	0.0
Experience with care	38.0	24.5	20.9	2.6
Advice from doctors and senior midwives	22.9	33.3	42.4	8.4
While enrolled as a midwife student	0.0	0.5	0.5	29.5
Schools that specialized in breastfeeding care	10.9	10.9	8.4	0.5
Never studied	0.0	0.5	2.1	10.5
Other	4.2	3.7	3.1	8.4

The study setting included midwifery facilities of 47 prefectures in Japan. The survey was conducted from 21 December 2023 to 28 January 2024.

### Most used methods for midwife diagnoses

First, the most commonly used methods for distinguishing between different mastitis types were calculated. The participants were more likely to ‘distinguish by breast massage’ (37.0%) and ‘use the Mastitis Care Flowchart 2020’ (38.0%). However, asking a doctor (6.8% and 1.0%) and not being aware of distinguishing between different mastitis types (9.4%) also showed values of approximately 10%. Table 3 summarizes the proportion of the most used methods for distinguishing between different mastitis types.

**Table 3:** The most used methods to differentiate between different types of mastitis by Japanese midwives (N=192)

*Methods*	*Percent*
Differentiate by breast massage	37.0
Use the Mastitis Care Flowchart 2020	38.0
Differentiate by ultrasound examination by a midwife	0.5
Differentiate by ultrasound examination by a doctor	6.8
Differentiate by milk culture by a doctor	1.0
Not aware of differentiating between different types of mastitis	9.4
Other	7.3

The study setting included midwifery facilities of 47 prefectures in Japan. The survey was conducted from 21 December 2023 to 28 January 2024.

Second, we calculated the proportion of the methods used most frequently by midwives in this study to diagnose mastitis. The most common method used was ‘medical history, visual examination, and palpation’ (65.6%). The next most common method was ‘two of the following: interview, visual examination, and palpation’ (13.0%); however, the difference from the most common method was large. The proportion of midwives who used breast ultrasound was very low, and they did not use it very often (1.6% and 0.5%). Midwives also did not frequently ‘ask a doctor to perform a breast ultrasound or milk culture’ (1.0%). Table 4 presents the proportion of methods midwives mostly use to diagnose mastitis.

**Table 4:** The most commonly used methods for diagnosing mastitis by Japanese midwives (N=192)

*Methods*	*Percent*
One of the following: medical history, visual examination, or palpation	8.3
Two of the following: medical history, visual examination, or palpation	13.0
Medical history, visual examination, and palpation	65.6
Use the ‘Flow of differential diagnosis’* in the mastitis care guidelines	7.3
Medical history, visual examination, palpation, and breast ultrasound	1.6
Breast ultrasound	0.5
Ask a doctor for a breast ultrasound or milk culture	1.0
Other	2.6

The study setting included midwifery facilities of 47 prefectures in Japan. The survey was conducted from 21 December 2023 to 28 January 2024.

* Flow of differential diagnosis: as shown in the Supplementary file.

### Most used methods for diagnosing lactational breast cancer

The most common method used by Japanese midwives to diagnose breast cancer in lactating women was ‘medical history, visual examination, and palpation’ (27.1%), followed by ‘size of the lump after milk removal or when the lump recurs’ (18.1%). Meanwhile, ‘ask other medical staff for a breast ultrasound or milk culture’ accounted for 23.9%. The rate at which midwives themselves performed breast ultrasound examinations was very low (3.2%). The most common methods used by Japanese midwives to diagnose breast cancer in lactating women are presented in Table 5.

**Table 5:** The most commonly used methods for diagnosing breast cancer during lactation by Japanese midwives (N=192)

*Methods*	*Percent*
One of the following: medical history, visual examination, or palpation	7.0
Two of the following: medical history, visual examination, or palpation	6.4
Medical history, visual examination, and palpation	27.1
Breast ultrasound by myself	3.2
Ask other medical staff for a breast ultrasound or milk culture	23.9
Size of the lump after milk removal or when the lump recurs	18.1
Using the ‘Flow of differential diagnosis’* route according the mastitis care guidelines	2.1
Other	2.6

The study setting included midwifery facilities of 47 prefectures in Japan. The survey was conducted from 21 December 2023 to 28 January 2024.

* Flow of differential diagnosis: as shown in the Supplementary file.

## DISCUSSION

This study shed light on how Japanese midwives could improve their practical skills in breast care after completing their formal education. The most used diagnostic methods were the subjective skills of interviewing, visual examination, and palpation. Mostly, midwives did not use medical equipment for diagnosing mastitis. Furthermore, midwives learned about breast care and mastitis through accumulated experience in breast care and from doctors and senior midwives. Knowledge about bacterial and severe mastitis was acquired through advice from doctors and senior midwives rather than from practical experiences. Midwives made judgments by consulting with others in the same profession regarding matters that were difficult to judge through practical experience. Midwives provided breast care without having learned about breast cancer from their student days when they obtained their midwifery qualifications, or without having learned about breast cancer at all.

### Actual state of midwives’ learning about breast care

First, gaining experience in clinical teams and learning from advanced-level seniors is said to be highly effective in education^14,15^; the participants in this study also often learned from doctors and senior midwives. Therefore, it was found that midwives learned effective breast care through experience gained through practice.

Second, knowledge about mastitis, particularly bacterial mastitis, which is particularly prone to becoming severe, was mainly obtained from advice from doctors and senior midwives and experience in breast care rather than from attending training sessions or learning at specialized schools for breastfeeding care. Moreover, it was thought that judging by seeing actual breasts would lead to a more accurate judgment.

Third, there were differences in the results regarding knowledge about severe mastitis, because midwives gained knowledge about severe mastitis through advice from doctors or senior midwives rather than through breast care experience. Regarding breast care experience, confusion is likely to occur in the process of making decisions about severe mastitis on one’s own, considering ways to deal with it, and encouraging care. It was found that when midwives are unsure about breast care decisions, they value consulting with their colleagues.

The midwives in this study belonged to midwifery clinics that focused on breast care or midwifery clinics that handled childbirths. They are thought to have more specialized knowledge about breasts than midwives who belong to hospitals. We asked the participants in this study about their knowledge of breast cancer during breastfeeding. Although the number of years of experience as midwives was approximately 30 years, most midwives had not learned about breast cancer since they entered school as midwife trainees. Although some midwives attended training sessions, the number was not high. Some study participants had never learned about breast cancer during breastfeeding. The incidence of breast cancer in young people is increasing worldwide^16^. A relationship exists between breast cancer onset and menopause^17^. This relationship may be because the incidence of breast cancer among young people in Japan is increasing; the age of onset of breast cancer is increasingly approaching the rising childbearing age. It can be said that midwives need to raise their awareness of the current situation in Japan, where the number of women with breast cancer is increasing, and that midwives need to learn about breast cancer. One of the reasons why Japanese midwives do not learn about breast cancer is thought to be the lack of opportunities. It is also thought that considering training sessions that allow midwives to deepen their breast cancer knowledge is necessary. Although the curriculum of midwife training schools in Japan stipulates that content related to breast care should be taught^18^, the regulations regarding specific content are at each training school’s discretion. The participants in this study had not had the opportunity to learn about breast cancer during lactation after they left training school. Breast cancer in breastfeeding women is often detected late, which increases the mortality rate^19^. This lack of experience may also impact the desire to learn. However, midwives making incorrect decisions in such situations owing to a lack of knowledge may lead to significant consequences faced by mothers.

### The most common method used by midwives to identify and diagnose breast-related issues

Although some midwives in this study did not distinguish between different types of mastitis, the most common method used by midwives to diagnose mastitis was ‘patient’s medical history, visual examination, and palpation’. This indicated that midwives did not use medical techniques to diagnose mastitis. Furthermore, they did not use ultrasound examinations to diagnose severe mastitis. There are also reports that Japanese midwives would like to use breast ultrasound examinations^12^. However, it was revealed that midwives judge the severity of mastitis using subjective techniques such as interviews, visual examinations, and palpation without using medical equipment. This study showed the high level of ability of Japanese midwives in breast care. However, delays in breast cancer treatment have also occurred due to midwives overlooking breast cancer during lactation. Passing on the breast care diagnostic techniques that midwives have traditionally used is important. However, midwives can also use medical equipment to make more accurate judgments without being too attached to tradition. This is thought to lead to early severe mastitis and breast cancer detection and treatment.

### Limitations

This nationwide survey was conducted in Japan. Although the survey was a nationwide one, no question on the region of Japan targeted was included, making it unclear which region’s midwives responded to the survey. Therefore, whether this was a nationwide survey is unclear, which is a limitation of this study. The number of study participants was smaller than the planned sample size. We suggest that this was because the survey was conducted two to three months before and after the earthquake and tsunami that occurred mainly in the Noto Peninsula, Ishikawa Prefecture, Japan. During the disaster, midwives throughout Japan provided support to mothers, children, and their families. However, the data from this study are valuable for the future education of midwives.

## CONCLUSIONS

In Japan, midwives learnt breast care methods while gaining experience in clinical practice. However, Japanese midwives had limited knowledge about breast cancer assessment. Furthermore, it was revealed that most midwives did not use medical equipment for breast assessments. This suggests that Japanese midwives had high breast care skills, but also suggests that by utilizing medical equipment, midwives may be able to assess breasts with greater accuracy than before.

## Supplementary Material



## Data Availability

The data supporting this research are available from the author on reasonable request.
