# Supplementary material for: Midwives’ knowledge and diagnostic practices for mastitis and breast cancer in breastfeeding women in Japan: A cross-sectional study
Source: Eur J Midwifery. 2025 Aug 29;9:10.18332/ejm/209494. doi: 10.18332/ejm/209494 (PMC12395512; doi:10.18332/ejm/209494)
Supplement: Supplementary file 1 [file EJM-9-38-s1.pdf]

４）鑑別診断の流れ (Flow of differential diagnosis)

問診、視診、触診から得られた情報をもとに、以下の流れに沿って問題の鑑別を行う。

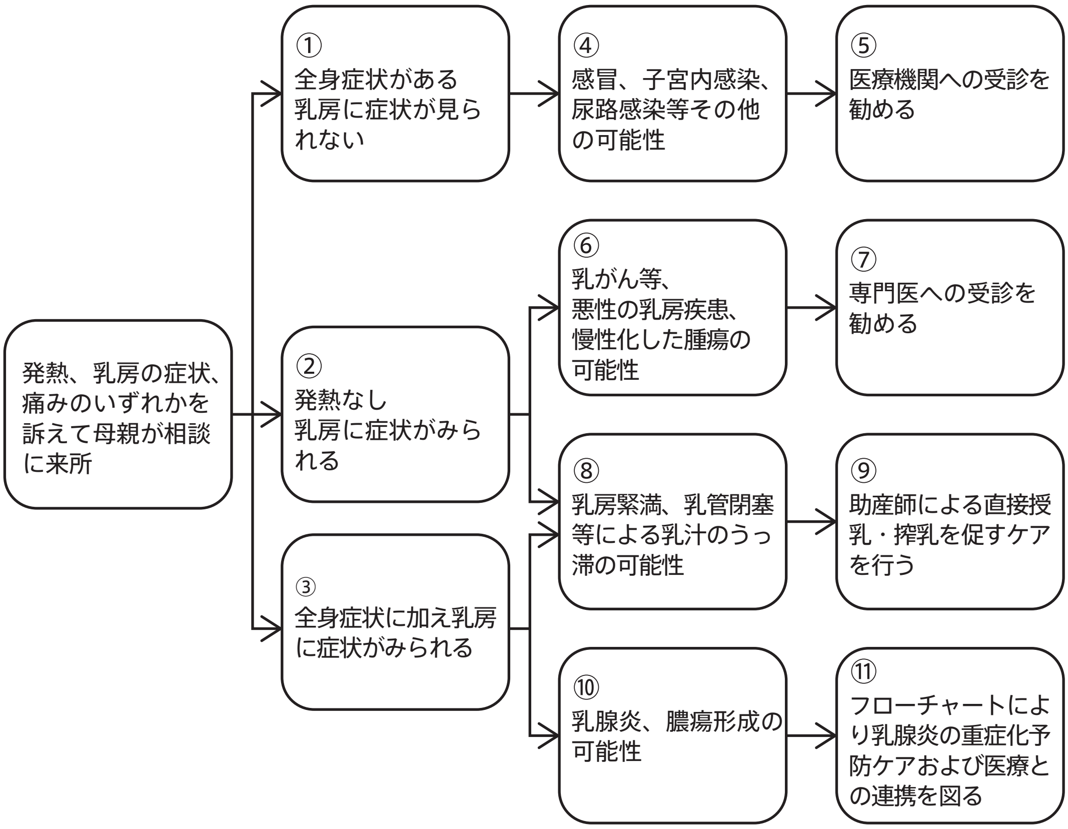

図１　発熱、乳房の腫れ、しこり、痛みを伴う母乳育児上の問題の鑑別フロー

(1)医療機関への受診を勧める場合

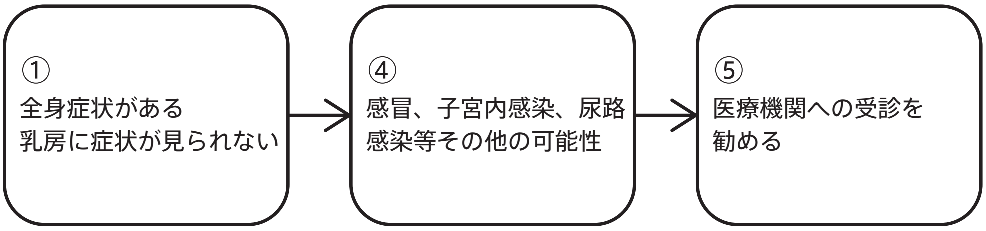

・発熱はあるが、乳房に症状が無い場合は、乳腺炎以外の疾患を疑い、医療機関への受診を勧める。
・発熱を伴う疾患は、風邪や尿路感染症、帯状疱疹、産褥早期では子宮感染などがある。まれに肋軟骨炎、膠原病や悪性腫瘍などの可能性もあるため、かならず受診するよう勧める。
・発熱時、検査や治療中の授乳は、ほとんどの場合継続は可能である。できる限り授乳が継続できるよう母親と保健医療者で協働する。

乳腺炎フローチャート2020（Mastitis Care Flowchart 2020）

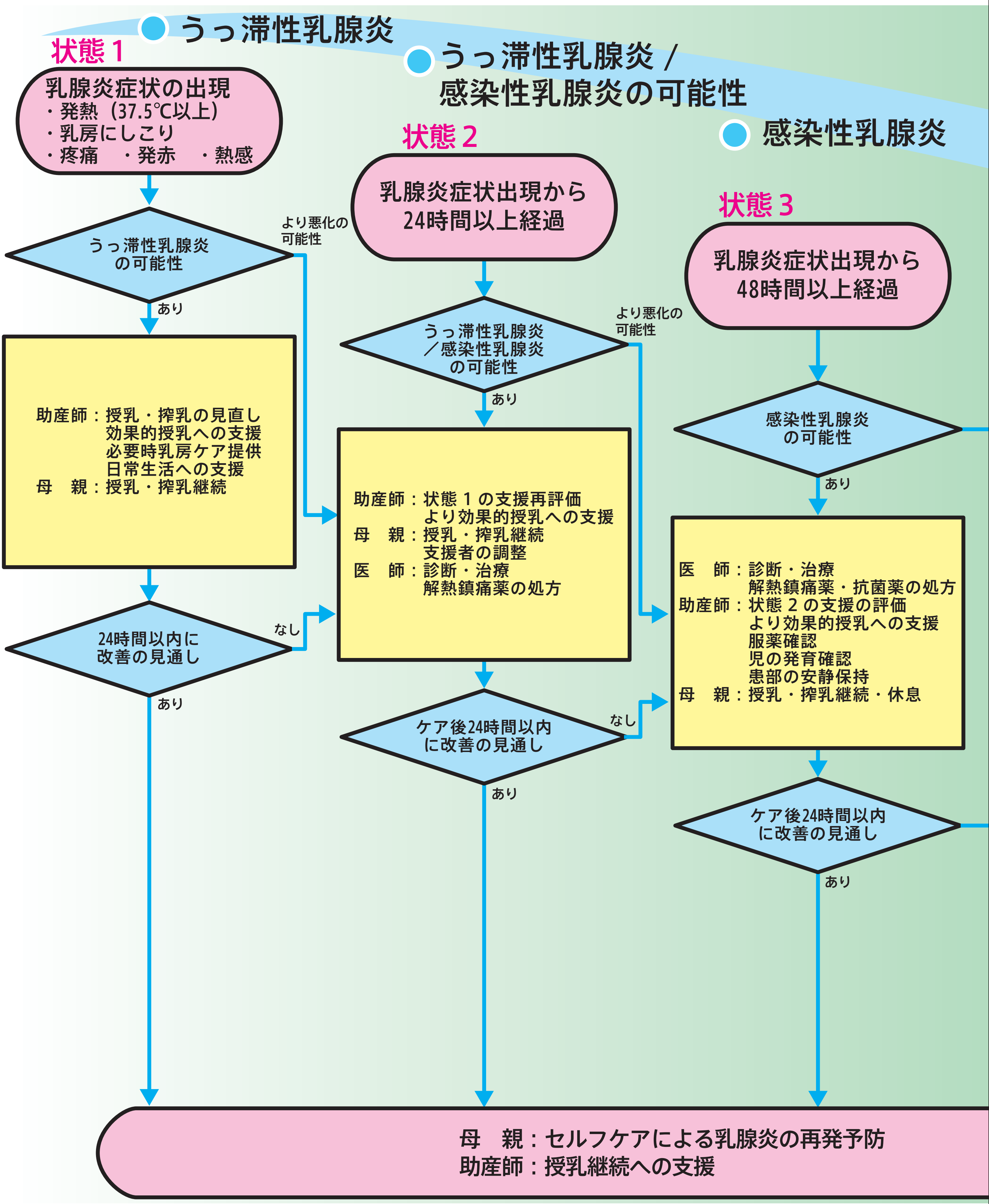

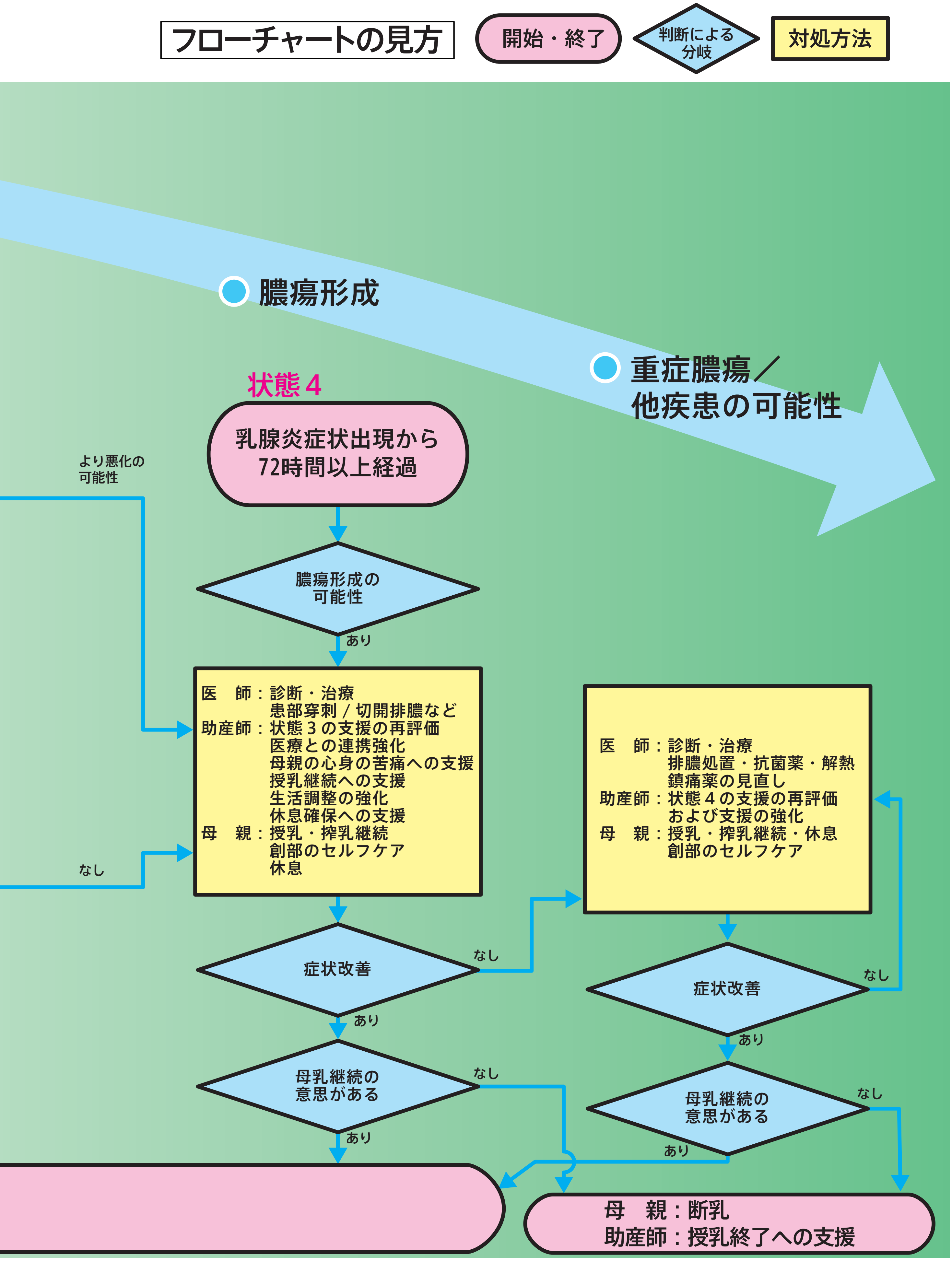

Japan Academy of Midwifery & Japanese Midwives Association. Mastitis Care Guidelines 2020 2nd edition, Japan Midwives Association, Tokyo. 2020. Accessed 15 November 2024.
https://www.midwife.or.jp/user/media/midwife/page/guilde-line/tab01/nyusenen_guideline_2020_2.pdf

Supplementary Material 2
Actual questionnaire used for "Midwives' knowledge and diagnostic practices for mastitis and breast cancer in breastfeeding women in Japan: A cross-sectional study"

「助産師による乳腺炎および乳がんの判断および対処方法の実態調査」に関するアンケート

[Survey on midwives' diagnosis and treatment of mastitis and breast cancer]

このアンケートの回答前に、必ず下記の２つの書類をお読みください。[Before answering this survey, please be sure to read the following two documents.]

① 研究協力依頼書 [Research collaboration request form]

② 研究内容説明書 [Research Description]

① および②の書類をお読みいただき、アンケートのご協力を頂けるようでしたら、下段の「□本研究に同意致します。」に☑をした上で、回答をお願い致します。[Please read documents ① and ② and if you would like to participate in the survey, the box below that says "□I agree to this research" before answering the question]

- 本アンケートは、５枚（２～６ページ）です。[This questionnaire is 5 pages long (2 to 6 pages).]
- 回答時間は、約１０分です。[Response time is approximately 10 minutes.]
- 回答後は、同封の回収封筒に入れて頂き、必ず封をお願い致します。[After answering your questions, please place them in the enclosed collection envelope and be sure to seal it.]

その後、お手数ですが、郵便ポストに投函をお願い致します。[After that, please post it in a mailbox.]

本研究に同意頂ける場合には、下記の□にチェックをお願い致します。[If you agree to this study, please check the box below.]

チェックがないアンケートは、同意なしとなり、回答が無効になりますので、ご注意ください。[Please note that surveys without a check mark will not be considered consent and your responses will be invalid.]

□ 本研究に同意致します。[I agree to this study.]

1． あなた自身の乳腺炎のケアに関してお答えください。[Please answer the following questions regarding the mastitis care you provide as a midwife.]

（1） あなたの乳腺炎(軽症～重症すべて含む)のおおよその対処数を、右下の□に記載してください。[Please write the approximate number of times you have treated your mastitis (including all cases from mild to severe) in the □ at the bottom right.]

1．(1) 約[Approximately] □ 名[menders]

（2） あなたの乳腺炎（軽症～重症すべて含む）の判断および対処方法について、あなた自身が学んだ中で<u>最も</u>学びが深まった方法に当てはまる項目を１つ選び、右下の□に項目の番号を記載してください。[Regarding how to diagnose and deal with mastitis (including all cases from mild to severe), please choose one item that you feel has taught you <u>most</u> and write the item number in the □ at the bottom right.]

① 書籍閲覧 [Reading book]
② 研修会参加 [Participating in training sessions]
③ Web サイト閲覧 [Browsing websites]
④ ケア経験 [During care experiences]
⑤ 医師や先輩助産師からの助言 [Advice from doctors or senior midwives]
⑥ 助産師学生在籍時 [While enrolled as midwifery students]
⑦ 母乳ケア専門の学校 [Schools specialising in breastfeeding care]
⑧ 学んだことがない [Never learned about it]
⑨ その他 [Other]（ご記入 [enter]：＿＿＿＿＿＿＿＿＿＿＿＿＿＿＿＿＿＿＿＿）

1．(2) □

（3） あなたの感染性乳腺炎のおおよその対処数を、右下の□に記載してください。[Please write the approximate number of cases of infectious mastitis you have treated in the □ at the bottom right.]

1．(3) 約[Approximately] □ 名[menders]

（4） あなたの感染性乳腺炎の判断および対処方法について、あなた自身が学んだ中で<u>最も</u>学びが深まった方法に当てはまる項目を１つ選び、右下の□に項目の番号を記載してください。[Please <u>most helped</u> you in learning how to diagnose and treat infectious mastitis, and write the item number in the □ at the bottom right.]

① 書籍閲覧 [Reading book]
② 研修会参加 [Participating in training sessions]
③ Web サイト閲覧 [Browsing websites]
④ ケア経験 [During care experiences]
⑤ 医師や先輩助産師からの助言 [Advice from doctors or senior midwives]
⑥ 助産師学生在籍時 [While enrolled as midwifery students]
⑦ 母乳ケア専門の学校 [Schools specialising in breastfeeding care]
⑧ 学んだことがない [Never learned about it]
⑨ その他 [Other]（ご記入 [enter]：＿＿＿＿＿＿＿＿＿＿＿＿＿＿＿＿＿＿＿＿）

1．(4) □

（5）　あなたの乳腺炎重症化者（排膿した方）のおおよその対処数を、右下の□に記載してください。[Please write the approximate number of people with severe mastitis (those with purulent discharge) that you have treated in the □ at the bottom right.]

１．（5）約[Approximately] □ 名[menders]

（6）　あなたの感染性乳腺炎の判断および対処方法について、あなた自身が学んだ中で最も学びが深まった方法に当てはまる項目を１つ選び、右下の□に項目の番号を記載してください。[Please most helped you in learning how to diagnose and treat infectious mastitis, and write the item number in the □ at the bottom right.]

① 書籍閲覧 [Reading book]
② 研修会参加 [Participating in training sessions]
③ Web サイト閲覧 [Browsing websites]
④ ケア経験 [During care experiences]
⑤ 医師や先輩助産師からの助言 [Advice from doctors or senior midwives]
⑥ 助産師学生在籍時 [While enrolled as midwifery students]
⑦ 母乳ケア専門の学校 [Schools specialising in breastfeeding care]
⑧ 学んだことがない [Never learned about it]
⑨ その他 [Other]（ご記入 [enter]：＿＿＿＿＿＿＿＿＿＿＿＿＿＿＿＿＿＿＿＿＿）

１．（6）□

（7）　あなたが行っている乳腺炎の判断方法で、最も当てはまる項目を１つ選び、右下の□に項目の番号を記載してください。[Please method you use to diagnose mastitis that best applies to you and write the number of the item in the □ at the bottom right.]

① 問診（痛み、倦怠感、発熱などの患者の訴え） [Medical history (patient's complaints of pain, fatigue, fever, etc.)]
② 視診（発赤などの乳房の状態）[Visual examination (breast conditions, such as redness)]
③ 触診（熱感、しこりなど触ることで分かる状態）[Palpation (conditions that can be palpated, such as heat, lumps, etc.)]
④ 問診（痛み、倦怠感、発熱などの患者の訴え）と視診（発赤などの乳房の状態）[Medical history (patient's complaints of pain, fatigue, fever, etc.) and visual examination (breast conditions, such as redness)]
⑤ 問診（痛み、倦怠感、発熱などの患者の訴え）と触診（熱感、しこりなど触ることで分かる状態）[Medical history (patient's complaints of pain, fatigue, fever, etc.) and palpation (conditions that can be palpated, such as heat, lumps, etc.)]
⑥ 視診（発赤などの乳房の状態）と触診（熱感、しこりなど触ることで分かる状態）[Visual examination (breast conditions, such as redness) and palpation (conditions that can be palpated, such as heat, lumps, etc.)]
⑦ 問診（痛み、倦怠感などの患者の訴え）、視診（発赤などの乳房の状態）および触診（熱感、しこりなど触ることで分かる状態）[Medical history (pain, fatigue, etc.) (patient complaints), visual examination (breast conditions such as redness), and palpation (conditions that can be palpated such as heat and lumps)]
⑧ 乳腺炎ケアガイドライン「鑑別診断の流れ」を用いる [Use of the "Flow of differential diagnosis" route in the Mastitis Care Guidelines]
⑨ 問診（痛み、倦怠感などの患者の訴え）、視診（発赤などの乳房の状態）、触診（熱感、しこ

りなど触ることで分かる状態）、および超音波検査 [Medical history (patient complaints of pain, fatigue, etc.), visual examination (breast conditions such as redness), palpation (conditions that can be palpated such as heat and lumps), and ultrasound examination]

⑩ 超音波検査 [Breast ultrasound examination]

⑪ 問診（痛み、倦怠感などの患者の訴え）と超音波検査 [Medical history (patient complaints of pain, fatigue, etc.) and breast ultrasound examination]

⑫ 医師に超音波検査を依頼 [Requesting a doctor to perform a breast ultrasound examination]

⑬ 医師に乳汁培養を依頼 [Requesting a doctor to perform a milk culture]

⑭ その他 [Other]（ご記入 [enter]：＿＿＿＿＿＿＿＿＿＿＿＿＿＿＿＿＿＿＿＿）

１．(7) □

（８） 乳腺炎と判断後、乳腺炎の種類を鑑別する方法で、最も当てはまる項目を１つ選び、右下の□に項目の番号を記載してください。[After determining that you have mastitis, choose the most applicable item to differentiate between different types of mastitis and write the item number in the □ at the bottom right.]

① 乳房マッサージによって鑑別する [Differentiate by breast massage]

② 乳腺炎ケアガイドライン「乳腺炎ケアフローチャート 2020」を用いる [Use the mastitis care guideline "Mastitis Care Flowchart 2020"]

③ 自ら超音波検査によって鑑別する [Differentiate by self-examination]

④ 医師の超音波検査によって鑑別する [Differentiate by ultrasound examination by a doctor]

⑤ 医師の乳汁培養によって鑑別する [Differentiate by milk culture by a doctor]

⑥ 乳腺炎の種類の鑑別は特に意識していない [Not particularly aware of differentiating between different mastitis types]

⑦ その他 [Other]（ご記入 [enter]：＿＿＿＿＿＿＿＿＿＿＿＿＿＿＿＿＿＿＿＿）

１．(8) □

（９） あなたは、医師の診察を依頼する前の段階で感染性乳腺炎の可能性があると判断するときに、どのように判断していますか？下記の□に、ご自由に記載をお願い致します。[How do you determine whether there is a possibility of infectious mastitis before consulting a doctor? Please feel free to write your answer in the □ below .]

2. あなた自身の授乳期乳がんの判断および対処方法について教えてください。[Please tell us about your own method of diagnosing and dealing with breast cancer while breastfeeding.]

（1） あなたの授乳期乳がんのおおよその対処数を、右下の□に記載してください。[Please write the approximate number of cases of breast cancer you have treated during lactation in the □ at the bottom right.]

2.（1）約[Approximately] □ 名[menders]

（2） 授乳期乳がんの判断および対処方法について、あなた自身が学んだ中で<u>最も</u>学びが深まった方法に当てはまる項目を１つ選び、右下の□に項目の番号を記載してください。[What do you the most important way to learn about how to identify and deal with breast cancer during lactation and write the item number in the □ at the bottom right.]

① 書籍閲覧 [Reading book]
② 研修会参加 [Participating in training sessions]
③ Web サイト閲覧 [Browsing websites]
④ ケア経験 [During care experiences]
⑤ 医師や先輩助産師からの助言 [Advice from doctors or senior midwives]
⑥ 助産師学生在籍時 [While enrolled as midwifery students]
⑦ 母乳ケア専門の学校 [Schools specialising in breastfeeding care]
⑧ 学んだことがない [Never learned about it]
⑨ その他 [Other]（ご記入 [enter]：＿＿＿＿＿＿＿＿＿＿＿＿＿＿＿＿＿＿＿＿）

2.（2）□

（3） あなたが行っている授乳期の乳がんの判断方法の<u>最も</u>当てはまる項目を１つ選び、右下の□に項目の番号を記載してください。 [Please <u>that best applies</u> to the method you use to determine breast cancer during breastfeeding , and write the item number in the □ at the bottom right.]

① 問診（しこり、違和感などの患者の訴え）[Medical history (patient's complaints of lumps, discomfort, etc.)]
② 視診（左右差などの乳房の状態） [Visual examination (condition of the breasts, such as differences between left and right)]
③ 触診（しこりなど触ることで分かる状態） [Palpation (condition that can be palpated, such as lumps)]
④ 問診（しこり、違和感などの患者の訴え）と視診（左右差などの乳房の状態）[Medical history (patient's complaints of lumps, discomfort, etc.) and visual examination (breast conditions, such as differences between left and right)]
⑤ 問診（しこり、違和感などの患者の訴え）と触診（しこりなど触ることで分かる状態）[Medical history (patient's complaints of lumps, discomfort, etc.) and palpation (condition that can be palpated, such as lumps)]
⑥ 視診（左右差などの乳房の状態）と触診（しこりなど触ることで分かる状態） [Visual examination (breast conditions, such as differences between left and right) and palpation (condition that can be palpated, such as lumps)]
⑦ 問診（しこり、違和感などの患者の訴え）、視診（左右差などの乳房の状態）および触診（しこりなど触ることで分かる状態） [Medical history (patient's complaints of lumps, discomfort, etc.), visual examination (breast conditions, such as differences between left and right), and palpation (condition that can be palpated, such as lumps)]
⑧ 問診（しこり、違和感などの患者の訴え）、視診（左右差などの乳房の状態）、触診（しこり

など触ることで分かる状態）および自ら超音波検査を実施 [Medical history (patient complaints such as lumps, discomfort, etc.), visual examination (breast conditions, such as asymmetry between the left and right breasts), palpation (conditions that can be palpated, such as lumps), and breast ultrasound examination]

⑨ 自ら超音波検査を実施 [Breast ultrasound examination]
⑩ 医師に超音波検査を依頼 [Requesting a doctor to perform a breast ultrasound examination]
⑪ 医師に乳汁培養を依頼 [Requesting a doctor to perform a milk culture]
⑫ 排乳後のしこりの大きさ [Size of the lump after milk removal]
⑬ しこりが繰り返されるとき [When the lump recurs]
⑭ 乳腺炎ケアガイドライン「鑑別診断の流れ」を用いる [Using the "flow of differential diagnosis" route in the mastitis care guidelines]
⑮ 産科医師や技師に超音波検査を依頼 [Requesting an obstetrician or technician to perform a breast ultrasound examination]
⑯ しこりがあるときには、どのような場合でも乳腺外来の受診をすすめる [When a lump is found, recommending a visit to a breast clinic in any case.]
⑰ しこりがあるときには、ご本人に乳がんのリスクについて説明する [When a lump is found, explaining the risk of breast cancer to the patient.]
⑱ その他 [Other]（ご記入 [enter]：＿＿＿＿＿＿＿＿＿＿＿＿＿＿＿＿＿＿＿＿）

2.（3）☐

3. あなた自身について、教えてください。 [Please tell us about yourself.]

（1） あなたの年齢を、右下の□に記載してください。 [Please enter your age in the ☐ at the bottom right.]

3.（1）☐歳 [years old]

（2） あなたの助産師経験年数を、右下の□に記載してください。 [Please enter the number of years of experience you have as a midwife in the ☐ at the bottom right.]

3.（2）☐年 [years] ☐カ月 [months]

（3） あなたの乳房ケア実施経験年数（母乳外来など一人で任されるようになった時点を開始と考えてください。）を、右下の□に記載してください。 [Please enter the number of years of experience you have providing breast care (starting from when they were entrusted with breastfeeding care alone, such as in a breastfeeding clinic) in the ☐ at the bottom right.]

3.（3）☐年 [years] ☐カ月 [months]

（4） あなたは、母乳ケアをどのような場所で学びましたか？ [Where did you learn about breastfeeding?]

① 助産師養成学校 [Midwifery School]

② 所属した病院 [Hospitals you worked at]
③ 桶谷式母乳管理法研修センター [Oketani Breast Milk Management Training Center]
④ ママズ乳房ケア(旧:すいな式乳房ケア)技術研修 [Mama's Breast Care technical training, or other)]
⑤ その他 [Other]（所属先記載 [(describe your affiliation)] ________________________）

3.（4）☐

（5） あなたの現在の所属先に当てはまる項目を選び、右下の□に項目の番号を記載してください。 [Please select the item that applies to your current affiliation and enter the item number in the □ at the bottom right.]

① 母乳専門の助産院 [Midwifery clinic specialising in breastfeeding]
② 分娩を扱う助産院 [Midwifery clinic that handles childbirth]
③ 産科（産婦人科）クリニックと助産院の兼業 [Concurrent obstetric clinic and midwifery clinic]
④ 総合病院(大学病院等含む)と助産院の兼業 [Concurrent general hospital and midwifery clinic]
⑤ 総合病院（大学病院等含む）[General hospital (including university hospitals, etc.)]
⑥ 産科（産婦人科）クリニック [Obstetric (gynecology) clinic]
⑦ その他 [Other]（所属先記載 [(describe your affiliation)] ________________________）

3.（5）☐

（6） 本研究に関して、乳腺炎や乳がんの判断や対処に関するご意見などがございましたら、下記の□にご自由にご記入ください。[If you have any opinions or comments regarding this study regarding the diagnosis and treatment of mastitis or breast cancer, please feel free to fill in the □ below.]

アンケートは、以上になります。[This concludes the survey.]

同意のチェックおよび各質問項目の回答忘れが無いかの最終確認をお願い致します。

[Please check that you have agreed and make a final check to make sure you have not forgotten to answer any questions.]

その後、専用の封筒に入れて、郵便ポストへの投函をお願い申し上げます。

[After that, please put it in the designated envelope and post it in a mailbox.]

ご協力ありがとうございました。[Thank you for your cooperation.]

© 2025 Kanazawa Y.
